# The false evidence rate: An approach to frequentist error rate control conditioning on the observed *P* value

**DOI:** 10.1073/pnas.2415706122

**Published:** 2025-01-10

**Authors:** Daniel J. M. Crouch

**Affiliations:** ^a^Centre for Human Genetics, Nuffield Department of Medicine, University of Oxford, Oxford OX3 7BN, United Kingdom

**Keywords:** *P* values, significance thresholds, hypothesis testing

## Abstract

A *P* value is conventionally interpreted either as a) the probability by chance of obtaining more extreme results than those observed or b) a tool for declaring significance at a prespecified level. Both approaches carry difficulties: b) does not allow users to make inferences based on the data in hand, and is not rigorously followed by researchers in practice, while (a) is not meaningful as an error rate. Although *P* values retain an important role, these shortcomings are likely to have contributed significantly to the scientific reproducibility crisis. We introduce the concept of defining long-run frequentist error rates given the observed data, allowing researchers to make accurate and intuitive inferences about the probability of making an error after proposing that the null hypothesis is false. As one approach, we define the false evidence rate (FER) as the probability, under the null hypothesis, of observing a hypothetical future *P* value providing evidence toward the alternative hypothesis suggested by the observed *P* value, which we define as a false positive. FERs are much more conservative than their corresponding *P* values, consistent with studies demonstrating that the latter do not effectively control error rates across the scientific literature. To obtain an FER below 5%, one needs to obtain a *P* value below approximately $5\times 10^{-5}$, while a *P* value of 5% corresponds to an FER of about 25%.

A common impulse when looking at data is to contemplate the probability of declaring that there is a true effect when the null hypothesis pertains: the probability of a false positive. However, such an exercise is fraught with difficulties in how to delineate data that are supportive of a true effect from those supportive of the null hypothesis. Under the Neyman–Pearson hypothesis testing framework, the solution is to specify a range of outcomes that has a low cumulative probability, α (usually 1% or 5%), under the null hypothesis, while maximizing statistical power (1), and the null hypothesis is rejected whenever the outcome falls within that range. While this approach does control the false positive rate (henceforth the “error rate”) in the long-run, over the entire population of *P* values that are generated, it does not describe the proportion of false positive results among the subset passing the α threshold, such as a set of results found in the scientific literature, which may as such contain a substantially higher proportion of false positives than α, because the proportion of studies with true nonnull effects is often low (2). A further limitation of the Neyman–Pearson framework is that it permits no information to be drawn from the exact *P* value, in the sense that the error rate α, which is specified before seeing the data, is the same regardless of whether *P* passes α only narrowly or by many orders of magnitude, even though the latter would be a stronger indication that the null hypothesis is false.

A common alternative approach is to interpret the *P* value as the conditional probability of observing future data that are, by chance, more extreme than the observed data, providing a rough guide to how unlikely the observed data would be if the null hypothesis was true. This was R.A. Fisher’s favored method for hypothesis testing (3). However, it is limited by its reliance on users’ intuitions about how these translate into error rates. Low *P* values, e.g., 0.01 can sound impressive in the Fisherian sense that there is a low chance of seeing results less likely than that under the null hypothesis, but the recent scientific replication crisis has demonstrated that error rates associated with *P* values of this magnitude are still quite high (4), and sampling variability in *P* values can be large (5, 6), so this approach may not always be reliable. Advantages of the Fisherian method are that it does not require a prespecified α value and that it allows the precise *P* value to be utilized.

In practice, researchers tend to use an informal blend of the Fisherian and Neyman–Pearson interpretations of *P* values, which may lead to additional confusion over their meanings (7). Further limitations to *P* values are that a) they do not provide quantification of the amount of evidence for the hypothesis in question, for example when comparing two *P* values to establish relative degrees of evidence, and b) it is not widely enough appreciated that *P* values narrowly passing the popular α thresholds 1% and, especially, 5%, often represent only weak evidence against the null hypothesis (2, 8). We address several aforementioned weaknesses of *P* values, by computing what the false positive rate would be for hypothetical *P* values observed in the future, if the researcher proposed that the null hypothesis was false based on their observed *P* value, which produces an error rate as does the Neyman–Pearson method, but also conditions on the observed *P* value as in the Fisherian method. This might be approached in a number of ways, but we will consider the probability of drawing data that provide further evidence for the alternative hypothesis ($H_{1}$), erroneously under the null hypothesis ($H_{0}$), after interpreting the observed *P* value $P_{\;\text{obs}}$ as being drawn from $H_{1}$. This can be pictured as hypothetical data being used in the future to test the proposition that $P_{\;\text{obs}}$ came from $H_{1}$, and our objective is to analyze what would be expected of these tests if $H_{0}$ was really true. As the hypothetical data are from $H_{0}$, evidence for $H_{1}$, if it were observed, would constitute an error. Interpreting $P_{\;\text{obs}}$ as being drawn from $H_{1}$ is equivalent to proposing that $H_{1}$ is true given $P_{\;\text{obs}}$, and so further evidence would support this proposition when it is false. It is assumed that the hypothetical future data have the same sample size as the data used to produce $P_{\;\text{obs}}$, as if one were repeating the same experiment or observational study. We quantify the evidence in favor of $H_{1}$, relative to $H_{0}$, from a hypothetical future *P* value $P_{\;\text{fut}}$ (not yet observed), while interpreting $P_{\;\text{obs}}$ as being drawn from $H_{1}$, as the likelihood ratio (LR):

[1] $$\;\text{LR}=\frac{L(\theta =\hat{\theta }\left|Z\left(P_{\;\text{obs}}\right))L(\theta =\hat{\theta }\right|Z\left(P_{\;\text{fut}}\right))}{L(\theta ={\hat{\theta }}_{\;\text{obs}}\left|Z\left(P_{\;\text{obs}}\right))L(\theta =0\right|Z\left(P_{\;\text{fut}}\right))},$$

where L is the normal likelihood function with mean θ and SD (σ) of 1, Z(P) is the Z-score corresponding to *P* value P, with smaller *P* values giving more positive Z-scores (using the inverse upper cumulative normal distribution), ${\hat{\theta }}_{\;\text{obs}}$ is the maximum likelihood estimate for θ when $Z(P_{\;\text{obs}})$ is the only variable from $H_{1}$, and $\hat{\theta }$ is the maximum likelihood estimate when both $Z(P_{\;\text{obs}})$ and $Z(P_{\;\text{fut}})$ are drawn from $H_{1}$. The numerator is the likelihood of a model in which $P_{\;\text{obs}}$ and $P_{\;\text{fut}}$ are both nonnull, while the denominator is the likelihood of a model in which $P_{\;\text{fut}}$ is null and $P_{\;\text{obs}}$ is nonnull. The likelihood ratio in Eq. **1** quantifies the relative evidence for $H_{1}$ versus $H_{0}$ from the future *P* value, based on the best available information (the estimates ${\hat{\theta }}_{\;\text{obs}}$ and $\hat{\theta }$), given that the observed *P* value was drawn from $H_{1}$. It thus measures evidence for $H_{1}$ provided by the future *P* value, after proposing that $H_{1}$ is true given only the observed *P* value. It is a meaningful likelihood ratio because the models in the numerator and denominator both have a single degree of freedom, allowing them to be fairly compared. We assume that $Z(P_{\;\text{obs}})$ and $Z(P_{\;\text{fut}})$ are normally distributed with SD 1. The evidence from $P_{\;\text{fut}}$ favors $H_{1}$ when LR>1, equivalently log(LR)>0, and each likelihood is normal with σ=1, thus the threshold above which $H_{1}$ is favored exists at:

[2] $$\begin{array}{cc} \;\text{log(LR)} & =\frac{-{\left(Z\left(P_{\;\text{obs}}\right)-\hat{\theta }\right)}^{2}-{\left(Z\left(P_{\;\text{fut}}\right)-\hat{\theta }\right)}^{2}}{2}\\ & \;+\frac{{\left(Z\left(P_{\;\text{obs}}\right)-{\hat{\theta }}_{\;\text{obs}}\right)}^{2}+Z{\left(P_{\;\text{fut}}\right)}^{2}}{2}\\ & =0, \end{array}$$

with the four terms being the exponents of four normal distribution functions, which are the four likelihoods in Eq. **1**, and the nonexponent parts of the normal functions, $1/\sqrt{2\pi }$, canceling. As $\hat{\theta }=(Z\left(P_{\;\text{obs}}\right)+Z\left(P_{\;\text{fut}}\right))/2$ and ${\hat{\theta }}_{\;\text{obs}}=Z\left(P_{\;\text{obs}}\right)$, Eq. **2** expands to

[3] $$\frac{Z{\left(P_{\;\text{fut}}\right)}^{2}}{2}+Z\left(P_{\;\text{obs}}\right)Z\left(P_{\;\text{fut}}\right)-\frac{Z{\left(P_{\;\text{obs}}\right)}^{2}}{2}=0,$$

and solving for $Z(P_{\;\text{fut}})$ gives^*^$Z\left(P_{\;\text{fut}}\right)=(\sqrt{2}-1)Z\left(P_{\;\text{obs}}\right)\approx 0.414\times Z\left(P_{\;\text{obs}}\right)$. We then compute the FER as the probability, under $H_{0}$, of $Z(P_{\;\text{fut}})$ exceeding this value, thus producing evidence to favor $H_{1}$:

[4] $$\;\text{FER}\left(P_{\;\text{obs}}\right)=1-\Phi (0.414\times Z\left(P_{\;\text{obs}}\right)),$$

where Φ represents the standard cumulative normal distribution, as $Z(P_{\;\text{fut}})$ is distributed as a standard normal variable under $H_{0}$. The FER is thus the probability of a randomly drawn Z-score from the null distribution exceeding a factor of 0.414 of the observed Z-score.^†^

$\;\text{FER}(P_{\;\text{obs}})$ gives the probability of observing further evidence in favor of $H_{1}$ under $H_{0}$, given $P_{\;\text{obs}}$. As evidence in favor of $H_{1}$ would be erroneous under $H_{0}$, the FER can be regarded as an error rate^‡^ associated with the proposal that $H_{1}$ is true based on $P_{\;\text{obs}}$. Upon computing an FER of 0.1, a researcher would be able to state: “if I proposed that $H_{1}$ were true, I would expect to produce evidence favoring $H_{1}$ 10% of the time, simply by chance.” The threshold for a satisfactory FER will depend on the context, including the cost associated with incorrectly producing evidence for $H_{1}$, and the prior evidence for $H_{1}$. However, it is reasonable to assume that low FERs, e.g., below 0.01 or 0.05, are very likely to be associated with true hypotheses, as they imply that the observed data point $P_{\;\text{obs}}$ was drawn from a model which evidence drawn from the null distribution rarely favors. A higher FER of, say, 0.4, would indicate that, after proposing that $H_{1}$ is true on the basis of $P_{\;\text{obs}}$, evidence in favor of $H_{1}$ when in fact $H_{0}$ is true would be seen 40% of the time, meaning there is an up to 40% chance of increasing the evidence for a false proposition,^§^ so the researcher should probably either disregard $H_{1}$, or regard it with greater suspicion than they did before observing $P_{\;\text{obs}}$. *P* values of 0.01 have FERs of 0.168, while *P* values of $5\times 10^{-5}$ have FERs of 0.054, illustrating the benefit of obtaining much smaller *P* values than 1%, in terms of ensuring much lower false positive rates as measured by the FER. A *P* value of 0.05 has a substantially higher FER of 0.248, which seems fairly consistent with the suggestion that 29% of published studies with P≈0.05 pertain to false hypotheses (9).

Fig. 1 shows the relationship between *P* values and their corresponding FERs, indicating how the rate of false evidence increases rapidly as *P* values rise from 0 to approximately 0.05. Curvature in the FER is minimized at $Z(P_{\;\text{obs}})\approx 1/0.414$, corresponding to $P_{\;\text{obs}}=7.86\times 10^{-3}$, which might serve as a useful demarcation point for classifying *P* values into those that provide lower vs. higher FERs (Fig. 1). This lies reassuringly close to the popular 1% α threshold.

**Fig. 1.**
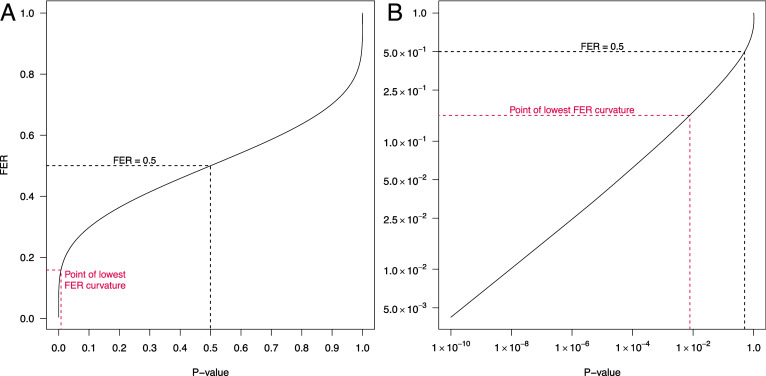
Relation between *P* values and their corresponding FERs: (*A*) standard scale and (*B*) log10 scale. Dashed lines show where P=0.5 corresponds to FER=0.5 (in black) and where the point of minimum curvature lies at $P\approx 7.86\times 10^{-3}$ (in red). Panel (*B*) provides better visualization for small *P* values.

An FER can be produced from a *P* value in a few steps, using any statistical software:

1. Compute the upper inverse cumulative normal distribution function (the upper normal quantile function) from the one-tailed *P* value. Two-tailed *P* values can be converted to one-tailed by either a) dividing by 2 when the effect observed in the study went in the direction expected under $H_{1}$ or b) dividing by 2 and subtracting this from 1, if the effect observed was opposite to that expected under $H_{1}$.
2. Multiply the resulting value by 0.414
3. Apply the upper cumulative normal distribution function to the result from (2).

In R, where P is the one-tailed *P* value, steps 1 to 3 above can be coded as:

pnorm(qnorm(P, lower.tail=FALSE)

* 0.414, lower.tail=FALSE).

The novelty of the FER lies in conditioning on the observed data while quantifying a long-run, frequentist error rate. Inference after conditioning is usually Bayesian, drawing on prior information about the model, but it is often not appropriate, or not appealing, to attempt to specify this information quantitatively. *P* value α thresholds, though requiring no such prior information, do not describe the error rate for a set of *P* values passing the threshold, or for a given individual *P* value, as they are designed to control an unconditional error rate; the long-run rate of errors over the full set of findings, both significant and nonsignificant, and not only the significant findings. The FER, in contrast, provides a separate error rate for each *P* value already observed, and thus circumvents this shortcoming of the Neyman–Pearson, α threshold, approach to *P* value interpretation, without requiring Bayesian assumptions. The FER does not render the Neyman–Pearson approach obsolete, as the latter allows researchers to control their unconditional long-run error rate using an α threshold, so long as this is specified before the *P* value is observed and followed correctly after observation. Instead, the FER provides a conditional error rate on the basis of the observed *P* value, which should serve as a more accurate guide to what researchers should conclude based on the results in hand. The α threshold may still take precedence, for example researchers may decide they cannot tolerate α higher than 1%, even if they would otherwise consider the FER from a higher *P* value, for example P=0.02 giving FER(0.02) = 0.20, to be an acceptable error rate for a given hypothesis of particular interest. But as the superior interpretative tool, FERs could be used to report results in manuscripts in place of α, with α instead reported like a data filtering parameter within the methods section. *P* values themselves may be reported alongside FERs, although there is a 1:1 relationship between the two (Fig. 1), if it is felt that readers might like to interpret them in the Fisherian way, but it should be made clear that it is the FER, and not the *P* value, quantifying a false positive error rate.

## Data Availability

There are no data underlying this work.

## Footnotes

^*^Another solution exists at $-(\sqrt{2}+1)Z\left(P_{\;\text{obs}}\right)\approx -1.414\times Z\left(P_{\;\text{obs}}\right)$, but this gives the threshold below which $Z(P_{\;\text{fut}})$ provides evidence that $H_{1}$ is true with an effect direction opposite to that suggested by $Z(P_{\;\text{obs}})$. We therefore do not incorporate this threshold into the FER calculation, although it could be used to produce a two-tailed version.

^†^When $P_{\;\text{obs}}>0.5$, the threshold $Z\left(P_{\;\text{fut}}\right)=0.414\times Z\left(P_{\;\text{obs}}\right)$ is negative, lying between $Z(P_{\;\text{obs}})$ and zero, so the FER becomes the probability of observing a future *P* value (drawn from the null) that either provides less evidence for $H_{1}$ than for $H_{0}$ (i.e., LR < 1), or, for very small future *P* values, evidence for $H_{1}$ with the opposite direction of effect to that suggested by $Z(P_{\;\text{obs}})$. However, it is still appropriate to regard this as the probability of an error, as if the researcher proposed a nonnull effect in the positive direction on the basis of $Z(P_{\;\text{obs}})<0$, they would continue to do so if they thought $Z(P_{\;\text{fut}})$ provided evidence of a null effect.

^‡^The long-run error rate is defined by the proportion $\lim_{n\to \infty }k/n$, where the random variable k is the number of errors and n is the number of trials. The error rate under the null after a single trial, proposing that the alternate hypothesis is true given $P_{\;\text{obs}}$, is 1/n=1 as only a single trial has been performed. Interpreting $\;\text{FER}(P_{\;\text{obs}})$ as the expected future error probability given $P_{\;\text{obs}}$, the long-run error rate becomes $\lim_{n\to \infty }(1+n\;\text{FER}\left(P_{\;\text{obs}}\right))/(1+n)=\;\text{FER}\left(P_{\;\text{obs}}\right)$.

^§^The FER provides the error rate assuming that $H_{0}$ is true, so it serves as an upper bound on the error rate in a similar way to an α threshold. As there is typically a nonzero prior probability that $H_{0}$ is false, the real error rate will usually be lower.
